# InChIKey collision resistance: an experimental testing

**DOI:** 10.1186/1758-2946-4-39

**Published:** 2012-12-20

**Authors:** Igor Pletnev, Andrey Erin, Alan McNaught, Kirill Blinov, Dmitrii Tchekhovskoi, Steve Heller

**Affiliations:** 1 Department of Chemistry, Lomonosov Moscow State University, 119991, Moscow, Russia; 2 InChI Trust, c/o FIZ CHEMIE Franklinstrasse 11, 10587, Berlin, Germany; 3 Advanced Chemistry Development, Inc. (ACD/Labs), 8 King Street East, Suite 107, Toronto, Canada, M5C 1B5; 4 Biomolecular Measurement Division, National Institute of Standards and Technology, Gaithersburg, MD, 20899-8362, USA

## Abstract

InChIKey is a 27-character compacted (hashed) version of InChI which is intended for Internet and database searching/indexing and is based on an SHA-256 hash of the InChI character string. The first block of InChIKey encodes molecular skeleton while the second block represents various kinds of isomerism (stereo, tautomeric, etc.). InChIKey is designed to be a nearly unique substitute for the parent InChI. However, a single InChIKey may occasionally map to two or more InChI strings (collision). The appearance of collision itself does not compromise the signature as collision-free hashing is impossible; the only viable approach is to set and keep a reasonable level of collision resistance which is sufficient for typical applications.

We tested, in computational experiments, how well the real-life InChIKey collision resistance corresponds to the theoretical estimates expected by design. For this purpose, we analyzed the statistical characteristics of InChIKey for datasets of variable size in comparison to the theoretical statistical frequencies. For the relatively short second block, an exhaustive direct testing was performed. We computed and compared to theory the numbers of collisions for the stereoisomers of Spongistatin I (using the whole set of 67,108,864 isomers and its subsets). For the longer first block, we generated, using custom-made software, InChIKeys for more than 3 × 10^10^ chemical structures. The statistical behavior of this block was tested by comparison of experimental and theoretical frequencies for the various four-letter sequences which may appear in the first block body.

From the results of our computational experiments we conclude that the observed characteristics of InChIKey collision resistance are in good agreement with theoretical expectations.

## Background

The International Chemical Identifier, InChI, is a unique representation of a chemical substance. InChI is developed under the auspices of IUPAC; it is free, and the source code of the InChI software is open. An InChI string is a sequence of characters derived from a structural representation of a substance, as its digital signature. The same string is always generated for any particular compound.

InChIKey is a 27-character compacted version of InChI which is intended for Internet and database searching/indexing and is based on an SHA-256 hash of the InChI character string.

It was introduced in 2007 with InChI Software release 1.02-beta. In 2009, the Standard InChIKey appeared with v. 1.02-standard release as an InChIKey computed from the Standard InChI; it is specifically designed for interoperability in exchange of chemical information. Since v. 1.03, InChI software has merged functionality allowing one to produce both Standard and Non-standard InChIKey (the current version of InChI Software is 1.04 , 2011 [1]). Note that the current format of InChIKey is different from that of the beta version (2007); the format of the Standard InChIKey is the same as that of v. 1.02-standard (2009).

An InChIKey string includes two hash blocks, three flag characters (which indicate whether the key is Standard; which version of InChI is used; and what is the protonation state of the molecule) and two separators (dashes).

The first hash block represents a molecular skeleton while the second block represents various additional kinds of isomerism (stereo, isotopic substitution, and, in the case of a Non-standard key, tautomerism). The first block is 14 characters long and the second block is 8 characters long; the characters are capital English letters.

The two blocks are character-encoded versions of bit strings which are hashes of the two substrings derived from a parent InChI. The hash function used for producing an InChIKey is the truncated cryptographic SHA-2 256-bit hash function [2]. The 65 and 37 bits of the full 256-bit SHA-2 signatures are retained for the first and the second block, respectively. Use of this cryptographic hash function (producing - hopefully - strongly randomized output) increases the chances that collision resistance will be as close to the theoretical limit as possible (see below).

InChIKey is designed to be a nearly unique representation of the parent InChI. However, a single InChIKey may occasionally map to two or more InChI strings (collision). It is noteworthy that the appearance of collision itself does not compromise the hashed representation as collision-free hashing is impossible in principle. The only viable approach is to set and keep a level of collision resistance regarded as reasonably sufficient for typical applications.

The practical goal of InChIKey design was to ensure a negligibly small probability of collisions for datasets of size characteristic of the largest available real-world molecular databases, ≈(50–100) × 10^6^ molecular skeletons, by guesstimate; for stereoisomers/isotopomers/tautomers, the practical goal was to avoid collisions up to several thousand isomers for a given molecular skeleton (plus of course some reservation for both blocks). Naturally, another design goal was to keep InChIKey reasonably short. All in all, this determined the choice of the length of hashes used in the first and the second blocks as 65 and 37 bits, respectively.

The estimated level of collision resistance was published when InChIKey was introduced in 2007. Despite the minor change in InChIKey layout upon transition from InChI Software v. 1.02-beta to v. 1.03 and further, this level remained the same and the same estimate was quoted with each new release [3]: “Note that due to the very essence of hash functions, collisions (the same InChIKey for different InChIs/structures) are unavoidable in very large collections. A theoretical – optimistic – estimate of collision resistance (i.e., the minimal size of a database at which a single collision is expected, that is, an event of the two hashes of two different InChI strings being the same) is 6.1 × 10^9^ molecular skeletons × 3.7 × 10^5^ stereo/isotopomers per skeleton ≈ 2.2 × 10^15^. To exemplify: the probability of a single first block collision in a database of 1 billion compounds is 1.3%. In other words, a single first block collision is expected in 1 out of 100/1.3 = 75 databases of 10^9^ compounds each. For 10^8^ (100 million) compounds in a database this probability is 0.014%.”

The section ‘Theoretical estimates’ below explains how the estimates of collision resistance for InChIKey first block (*6.1 × 10*^*9*^*molecular skeletons*) and second block (*3.7 × 10*^*5*^*stereo/isotopomers per skeleton*) are obtained using probability theory.

The tests which preceded the release of InChIKey in 2007 demonstrated the absence of collisions for several multi-million-record databases, up to a total size of ≈ 77 × 10^6^ molecules.

Nevertheless, it was and is evident that the potential size of chemical space greatly exceeds the capacity of InChIKey so collisions are unavoidable in large enough collections. And, of course, collisions are even more likely in computer-generated molecular libraries which may be made nearly as large as desired.

To illustrate this, it is probably enough to say that the number of constitutional isomers (*i.e.*, molecular skeletons) for the alkanes C_30_H_62_ is ≈ 4 × 10^9^ while for C_50_H_102_ it is ≈ 1 × 10^18^[4]. That is, the limits of InChIKey’s first block collision resistance should be nearly reached upon enumerating isomers of triacontane, while pentacontane lies far beyond these limits.

In 2009, collisions were reported for the second block of InChIKey [5]. The collisions were obtained for stereoisomers of Spongistatin I, a complex molecule containing 26 stereogenic elements (see below for more details on Spongistatin I).

In 2011, there appeared a report on a collision for the first block of InChIKey [6]. The two collided molecules are an alcohol and a ketone containing long branched alkyl radicals, C_50_ and C_56_, respectively.

In both reported cases, no details were published on how the collisions were obtained -- in particular, whether they occurred by chance or resulted from dedicated computational effort -- or on whether the collided structures exist in real-world chemistry.

Though collisions are indeed expected for molecules of such complexity as cited above, these reports prompted us^a^ to investigate, in more detail, how InChIKey’s collision resistance fits the theoretical probabilistic estimate. We computed related values in computational experiments, for the first and the second block of InChIKey separately, and compared them to theory.

The conclusion is that the observed values are in good agreement with theoretical ones (see below); that is, InChIKey behavior fits expectations.

### Hashing: probability theory and estimate of collision resistance

Collision occurs when two different values have the same hash code. As any hash function maps input values to more compact space, collisions are unavoidable and the only valid question is how often they appear, not if they appear at all.

Evidently, the best collision resistance would be achieved if hash values were ideally random and followed uniform distribution (that is, if producing any hash value for arbitrary input was equally probable). Of course, this ideal is impossible as pure true randomness can not be obtained using a deterministic hash function. Anyway, the best-case estimates of collision frequency may be drawn by analyzing uniformly distributed random variables.

Estimation of a theoretically expected number of collisions -- at given hash length and given dataset size -- is closely related to a well-known problem from probability theory, the *birthday paradox*[7]. This paradox is that the probability of finding two people with the same birthday in, say, a room at a party is counter-intuitively high. Thus, with probability > ½, 23 people are enough to find a same-birthday pair of persons (i.e., a birthday collision). If there are 28 people, the expected value for the number of same-birthday pairs is nearly 1.

Note that the mapping person - > birthday is fully analogous to hashing a dataset of *k* inputs – individual persons – to *k* outputs which belong to the fixed interval of *p* possible values, *p* = 365; that is, we reduce the potentially huge variety of individuals to a fixed 365-entry set of birthdays.

In fact, the *birthday paradox* is not a paradox at all, as the facts fully conform to the rules of probability theory [7,8]. Assuming that output values of hash functions are distributed in a perfectly uniform manner, one may estimate the probability of having collisions for a given number of input entries in a dataset, *k* (in our example, *k* is the number of people in the room). According to [8], the probability that all output (hashed) values are distinct is at most ½ when

(1) $$k\geq \left[1+\mathrm{sqrt}\left(1+\left(8\ln2\right)p\right)\right]/2$$

(the probability further decreases when *k* increases).

For *p* = 365 days in year, this translates to the number of peoples *k* ≥ 23.

Going with the formula (1) from birthdays to InChIKeys, one may easily calculate that for a 37-bit hash, *p* = 2^37^, half a chance of the presence of collisions is reached for a dataset containing ca. 4.37×10^5^ entries. For a 65-bit hash, *p* = 2^65^, it is reached for a dataset of ca. 7.15×10^9^ entries (the exact values are 436499 and 7151589655, respectively.).

Alternatively, we may use a slightly different formulation and – instead of probabilities - estimate the expected number of collisions for datasets of any given size (this expected value may be conveniently compared further to experimentally observed average).

Following [8], consider an indicator random variable *X*_*ij*_ which is 1 if persons *i* and *j* (of total *k*) have the same birthday and 0 otherwise. If birthdays are uniformly distributed among *p* = 365 possibilities, for any pair the probability of same birthday is 1/*p* = 1/365; the expected value (mathematical expectation) of *X*_*ij*_ is, by definition, D(*X*_*ij*_) = 1*(1/*p*) + 0*(1-1/*p*) = 1/*p*.

Consider *X* = ∑*X*_*ij*_ over all pairs *i*,*j*. Its expected value D(*X*) is also an expected value for the number of same-birthday pairs, or collisions^b^. Evidently, D(*X*) is the sum of D(*X*_*ij*_) over all pairs *i*,*j*, that is

(2) $$D\left(X\right)=K\left(k-1\right)/2\times \left(1/p\right)=k\left(k-1\right)/\left(2p\right)$$

If *k* = 23 (persons), and *p* =365 (days), the expected number of birthday collisions D(*X*) is 0.69. At *k* = 28 persons, D(*X*) is 1.04.

Now we go from birthdays to InChIKeys with the formula (2). For the first block, the hash length is 65-bit so *p* = 2^65^. Then the expected number of collisions for the dataset of *k* = 1 × 10^9^ entries (InChIs) D(*X*) = 0.0136, while for *k* = 1 × 10^10^ entries (InChIs) it is 1.3553.

The expected value of ½ for collision number, D_1_ = 0.5 (subscript 1 signifies first block), is reached at *k*_*1*_^0.5^ ≅ 6.1 × 10^9^ input entries (i.e. InChIs with different first block).

For the second block, the hash length is 37-bit, *p* = 2^37^. The expected number of collisions for a dataset of *k* = 1 × 10^5^ entries (InChIs) is 0.0320, while for *k* = 1 × 10^6^ entries (InChIs) it is 3.6380. The expected value of ½ for collision number, D_2_ = 0.5, is reached at *k*_*1*_^0.5^ ≅ 3.7 × 10^5^ input entries (i.e. InChIs with different second block).^c^

Exactly these values of *k*^0.5^, 6.1 × 10^9^ and 3.7 × 10^5^, are quoted in InChI documentation as InChIKey collision resistance for the first and the second blocks, respectively.

### InChIKey collision resistance: testing the second block

To check practical collision resistance of the relatively short 37-bit second block of InChIKey, we used the simple protocol of exhaustive direct testing. First, we generated all stereoisomers of a complex molecule which has so many stereogenic elements that expectation of second block collisions is high. Then we generated respective InChIs and InChIKeys and selected InChIKey subsets of various size *k*, up to the size of the whole set. Then, for each subset, computed the number of collisions *N*_2_ (subscript 2 stands for the second block; of course, the first block is the same for all isomers) and compared it to the theoretically expected value D_2_(*k*). Of course, for each given size *k* of subset, *m* multiple random samplings were performed and respective collision numbers were averaged.

To generate stereoisomers (as individual MOL files), we used a custom-written Python script. InChI and InChIKey were generated from these MOL files with inchi-1 executable v. 1.04, 64-bit version for Linux [1]. Random permutations of InChIKey subsets of various sizes were produced with the Unix *shuf* utility.

An obvious choice of test molecule was Spongistatin I for which collisions in the InChIKey second block had been previously reported [5].

**Figure 1:**
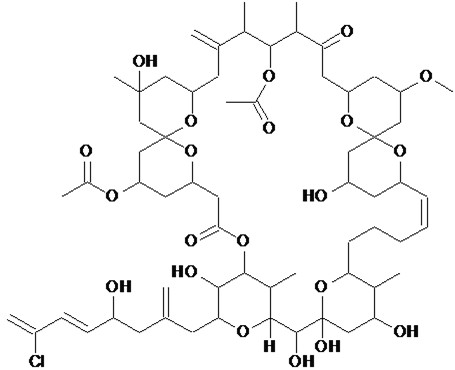
Molecular skeleton of Spongistatin I.

Spongistatin I is a complex molecule comprising 24 tetrahedral stereocenters and 2 stereogenic double bonds, Figure 1 (for a review of related chemistry, see [9]). The full set of its stereoisomers comprises 2^26^ = 67,108,864 structures (evidently, many of them are purely theoretical owing to significant steric strain).

**Figure 2:**
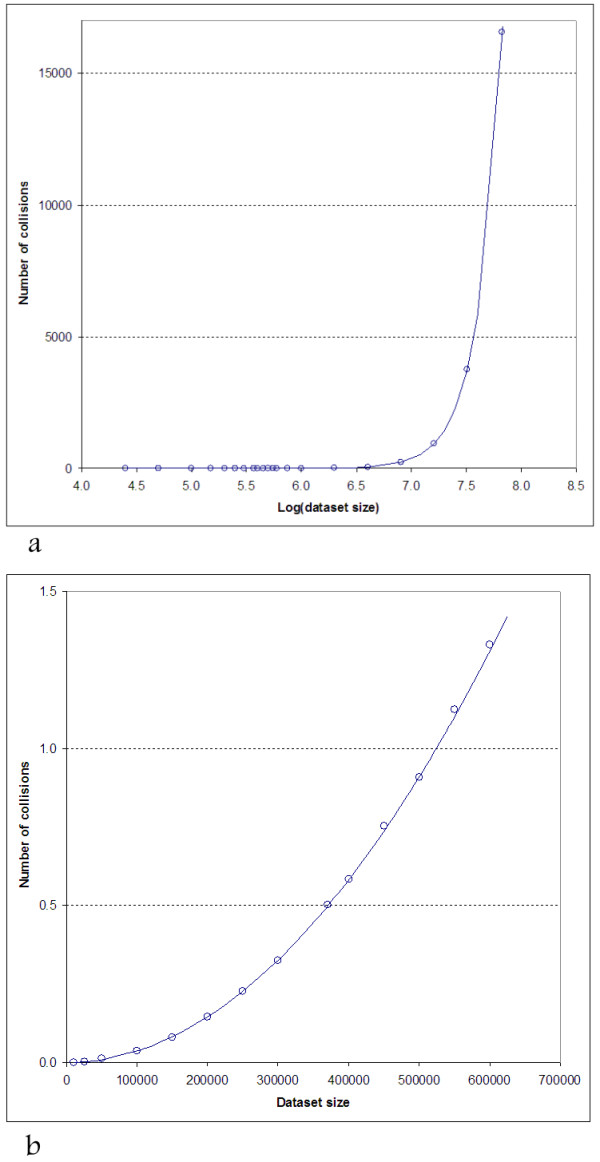
**The observed (circles) and theoretically expected (curve) average number of InChIKey second block collisions vs. the number of considered stereoisomers of Spongistatin I.a**) The whole data range; abscissa values: log(number of isomers); **b**) low-collision region; abscissa values: number of isomers.

The representative observed numbers of collisions are listed in Table 1 alongside with those theoretically expected for a 37-bit hash. Also, more data points are presented in Figure 2a (all results, logarithmic scale for dataset size) and 2b (the most practically important case of subsets with collision number less than or close to 1).

**Table 1:** Comparison of observed and theoretically expected average numbers of InChIKey second block collisions for stereoisomers of Spongistatin I

**Number of isomers,*k***	**Expected value of the number of collisions for a 37-bit hash, D**_**2**_	**Observed average number of collisions,*N***_**2**_	**Number of random samplings,*m***
10000	0.0004	0.0004	10000
50000	0.0091	0.0125	10000
100000	0.0364	0.0364	10000
250000	0.2274	0.2264	10000
**370000**	**0.4980**	**0.5011**	**10000**
500000	0.9095	0.9091	10000
1000000	3.6380	3.6390	10000
2000000	14.5519	14.6810	1000
4000000	58.2076	58.6810	1000
8000000	232.8306	234.9040	1000
16000000	931.3225	941.2460	1000
32000000	3725.2902	3766.7530	1000
**67108864**	**16383.9998**	**16565.0000**	**1**

As is seen, the observed and theoretical values do agree well.

In particular, the average number of collisions for the 10 000 randomly sampled datasets containing 370 000 entries each is 0.501 (Table 1) which is close to the theoretical value of 0.498.

Note that a collision number of ≈ 0.5 does not mean that half of the individual datasets have no collisions while the other half show exactly one collision. The distribution is more complex: in the particular random sampling of 10 000 datasets, 6 050 demonstrated no collisions, 3 056 gave a single collision each, 740 gave 2 collisions each, 142 gave 3, 11 gave 4, and 1 gave 5 collisions.

**Figure 3:**
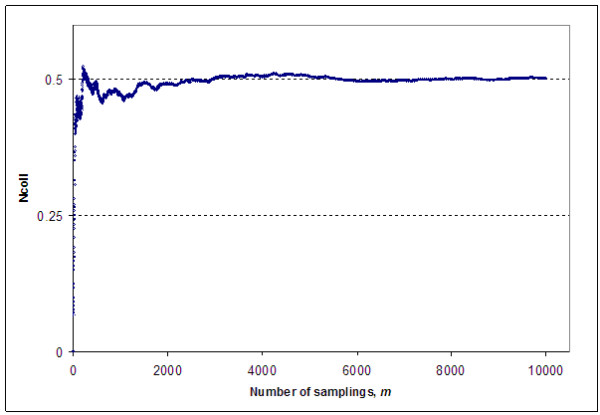
The dependence of observed average number of InChIKey second block collisions for 370 000-entry datasets vs. the number of samplings m.

Figure 3 shows how the average number of collisions observed for randomly sampled 370000-entry datasets changes with increase in number of samplings; it finally reaches a plateau close to 0.5 (this graph confirms the robustness of the observed average).

### InChIKey statistical behavior: testing the first block

The first block of InChI key is much longer than the second one so testing this block requires some additional effort/tricks.

The first evident problem concerns the source of the very large number of chemical structures which are necessary to generate the wide variety of [the first block of] InChIKeys. No existing database in public access contains more than a hundred million structures, which is evidently not enough for reliable tests of collision resistance of the first block. Thus, we chose to generate the necessary structures algorithmically.

For this purpose, we employed a specialized version of a structure generator [10] which is a part of the ACD/Structure Elucidator software [11-13] and is capable of generating all constitutional isomers corresponding to a given molecular formula. Feeding this software with various molecular formulae allowed us to gather as many non-equivalent structures as necessary (for an example of molecular formulae used with counts of generated structures, and a structure generation software screenshot, see Additional file 1: Table S1 and Additional file 1: Figure S1).

The InChIKeys were generated using InChI Software 1.04 [1]. The structure generator does not produce duplicated structures but does not recognize possible tautomers which can produce the same InChIKeys. Generation of tautomers was avoided by appropriate selection of molecular formula. The generated InChIKeys were stored to file for further analysis with PowerGREP software [14].

The other problem is related to difficulties in the handling huge amounts of data obtained in computational experiments. According to the theory given above, the requirement for a direct observation number of, say, 100 collisions for the first block is achieved for a set of about 1 × 10^11^ InChIKeys which would require 3 terabytes just to store the keys. Generation and searching for non-unique keys in such a huge dataset is a challenging task even for powerful computers. In addition, the possible equivalence of Standard InChIKeys for different InChIs which arise from tautomerism makes necessary the storage of additional identifiers (e.g., original InChI alongside InChIKey) and significantly increases the amount of necessary storage space.

Thus, direct testing of collision resistance for the 65-bit/14-letter first block (identical to that performed for the shorter second block) is an extremely resource-demanding task that we could not afford during this work.

The tests described below are indirect. Instead of checking how close is the behavior of the whole 14-letter string to uniform random distribution, we tested the randomness and uniformity of appearance for various short sub-blocks of the first block. If this hypothesis is confirmed, it is a strong though indirect proof that the statistical properties of the whole first block (and thence its collision resistance) match the theoretical expectations.

A specific precaution is necessary due to the fact that the number of unique values for the 65-bit first block *p* is 3.69 × 10^19^ (2^65^) while the number of unique 14-letter strings is 6.45 × 10^19^ (26^14^). This means that some sequences of 14 letters may not constitute a valid first block of InChIKey. That is, theoretically expected frequencies of appearance of specific letters and letter sequences should be calculated accounting for the internal layout of the InChIKey string.

**Figure 4:**
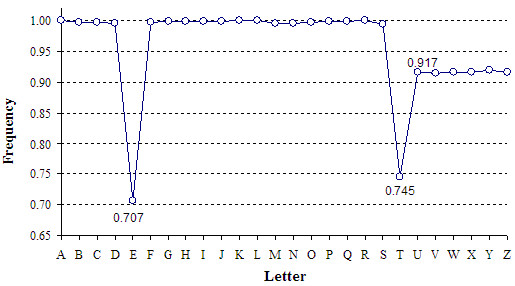
**Normalized frequencies of various letters within the first block of InChIKey.** Measured using InChIKeys for 1 097 996 constitutional isomers of C_8_H_8_Cl_3_F_5_; the values are normalized to the frequency of ‘A’.

To illustrate, let us consider the experimental letter frequencies inside the first block of InChIKey which we obtained using a set of about 1 × 10^6^ InChIKeys. As shown in Figure 4, the frequencies for various letters do differ. In particular, ‘E’ and ‘T’ are relatively rare; also, the letters ‘U’ to ‘Z’ appear 10% less frequently than most of the others.

An investigation of letter frequencies for specific position inside the first block reveals the detailed pattern(s); see Table 2 which lists the selected data (for a non-truncated version of the table see Additional file 1: Table S2). Shown in bold are the values which significantly deviate from a typical one.

**Table 2:** Selected normalized letter frequencies for various positions (1 to 14) in the InChIKey first block

**Position**	**1**	**2**	**3**		**4**	**5**	**6**	**7**	**…**	**12**	**13**	**14**
**A**	1.0000	0.9221	0.9130	0.9917	0.9213	0.9227	1.0039		0.9250	1.2273	0.9390
**…**	…	…	…		…	…	…	…	…	…	…	…
**D**	0.9898	0.9223	0.9182	1.0038	0.9138	0.9260	0.9938	…	0.9291	1.2321	0.9380	
**E**	**0.0000**	0.9164	0.9331	**0.0000**	0.9208	0.9256	**0.0000**	…	0.9216	1.2249	0.9436	
**F**	0.9986	0.9145	0.9200	0.9964	0.9183	0.9283	0.9957	…	0.9171	1.2313	0.9430	
**…**	…	…	…		…	…	…	…	…	…	…	…
**R**	0.9965	0.9109	0.9279	1.0015	0.9207	0.9269	0.9916	…	0.9213	1.2152	0.9420	
**S**	0.9940	0.9186	0.9294	0.9956	0.9184	0.9259	0.9957	…	0.9226	1.2236	0.8924	
**T**	**0.2395**	0.9237	0.9303	**0.2351**	0.9227	0.9291	**0.2297**	…	0.9268	**0.8413**	0.9037	
**U**	0.9992	0.9573	0.9341	0.9934	0.9543	0.9303	0.9906	…	0.9251	**0.0000**	0.8902	
**…**	…	…	…		…	…	…		…	…	…	…
**Z**	0.9986	0.9627	0.9293	0.9969	0.9569	0.9288	0.9873	…	0.9316	**0.0000**	0.8857	

Measured using InChIKeys for 998753 isomers of C_9_H_8_O_1_; the values are normalized to the frequency of ‘A’.

One may see from these data that the whole 14-letter string of the first block is composed of four 3-letter substrings (triplets, each has the same internal distribution of letters) followed by a 2-letter substring (doublet).

It is possible to determine which letter sequences may occur within the triplets and the doublet. An each triplet may be a sequence “AAA” through “ZZZ” -- except for “EAA” to “EZZ” and for “TAA” to “TTV” (the listing follows lexicographical order changing the rightmost letters first, e.g., “AAA”, “AAB”, “AAC”, … ). This amounts to a total of 16384 possible letter sequences. The valid sequences for a doublet are “AA” to “TR”; the total is 512. The grand total for the first block is thus 3.69 × 10^19^ (16384^4^ × 512) possible 14-letter sequences which fits the number of unique values for a 65-bit hash, *p* = 2^65^.

Note that the 3-letter sequences which start from ‘E’ (and most of those which start from ‘T’) may not occur within the triplets. Also, no 2-letter sequences which start with letters from ‘U’ to ‘Z’ may occur within the doublets. These rules explains the low frequency of letters “E’,’T’, ‘U’-‘Z’ observed in InChIKeys, in general (Figure 4) and in specific positions (Table 2).

This specific layout of the first block directly reflects the encoding schema chosen in current InChI Software to represent a 65-bit binary hash with 26 Latin letters. The same schema is used for the second block (it is composed of 2 triplets and a terminal doublet). Of course, the layout might have been revealed through digging into source code; however, the “black-box” analysis shown above parallels the whole “experimentalist” design of this study; also the letter frequencies obtained indirectly support the random character of InChIKey strings.

Now when the layout is established, one may easily calculate the theoretical probability of appearance of any *s*-letter sequence at any position of the first block of InChIKey. Comparing them with those obtained in the computational experiment may confirm or disprove the uniform random character of InChIKey.

By balancing the length of investigated letter sequences against computational feasibility, we chose investigation of 4-letter sequences (technically, all the generated InChIKeys were checked for the presence of predefined 4-letter sequences and only those passing the check were stored for further analysis).

We calculated the probabilities associated with all the possible 4-letter sequences. Assumption of a uniform random distribution means that all valid letter sequences for triplets and doublet are equally likely and that the content of any triplets/doublet is independent of the others. That is, for example, the probability *P* of the presence of the letter segment “ABCD” at the first position of the first block of InChIKey, can be calculated as follows:

(3) $$P=\left(P\mathrm{of}“\mathrm{ABC}”{\mathrm{in}\;1}^{\mathrm{st}}\mathrm{triplet}\right)\times \left(P\mathrm{of}‘D’{\mathrm{at}\;\mathrm{the}\;\mathrm{first}\;\mathrm{position}\;\mathrm{of}\;2}^{\mathrm{nd}}\mathrm{triplet}\right)=\left(1/16384\right)\times \left(26/512\right)=2.5183\times 10^{-6}$$

Calculating and summing the probabilities for all 11 possible positions of the 4-letter substring inside the first block one may find the final probabilities for the specific letter sequences to appear in the first block (accounting for the above described layout which limits appearance of some letters in some positions).

It is easy to convert these probabilities to theoretically expected numbers of occurrence of different letter sequences. Table 3 provides the related data for 16 4-letter sequences of four kinds which differ in starting letter and thus in associated probability. The theoretical values are accompanied by experimentally obtained ones.

**Table 3:** The occurrence of various 4-letter sequences in the set of first blocks for 1.2002 × 10^9^ InChIKeys

**Sequence**	**Experiment**	**Theory**	**Ratio**		**Sequence**	**Experiment**	**Theory**	**Ratio**
ABCD	33243	33041	1.006		EDNA	20135	20254	0.994
FMGL	33389	33041	1.011		EGPS	20466	20254	1.010
LGRC	32793	33041	0.992		EKPH	20344	20254	1.004
RBCQ	32937	33041	0.997		EJDO	20190	20254	0.997
Probability		2.7530 × 10^-5^		Probability		1.6876 × 10^-5^		
TBAC	20209	20254	0.998		ZAMR	33551	33547	1.000
TKIL	20303	20254	1.002		ZDKL	33650	33547	1.003
TRPC	20273	20254	1.001		ZSBC	33581	33547	1.001
TSBF	20111	20254	0.993		ZIII	33577	33547	1.001
Probability		1.6876 × 10-5		Probability		2.7951 × 10-5		

The ratio of experimental and theoretical occurrences ranges from 0.99 to 1.01 and is very close to the ideal value of 1 (standard deviation for this ratio is 0.0055).

We also performed additional tests with 3-, 4-, 5- and longer-letter sequences employing different sets of chemical structures; more than thirty billion (3 × 10^10^) InChIKeys were generated during this work.

All the collected data demonstrate good agreement between experiment and theory. This indirectly confirms the theoretically expected uniform random distribution of InChIKey first block values, which in turn supports the declared collision resistance.

## Conclusions

Combining the results of computational experiments for the first and the second blocks, we conclude that the observed statistical characteristics of InChIKey collision resistance are in good agreement with theoretical expectations. Of course, the collision resistance may be further improved by employing longer hashes; however, the current design and implementation seem to meet their goals.

## Endnotes

^a^The present study extends/updates our tests of InChIKey collision resistance performed in 2007–2009 which remained mainly unpublished. Also, some preliminary results concerning the first block were reported at the 2012 ACS Spring Meeting.

^b^Strictly speaking, the number of same-hash pairs of input values is slightly different from the number of collisions, i.e., ‘already seen’ hash values. The numbers are exactly equal if no composite collisions (i.e., 3 or more inputs mapped to the same output) occur.

^c^More precisely: D_1_(6.1 × 10^9^) =0.504; D_2_(3.7 × 10^5^) =0.498*.*

## Competing interests

The authors declare that they have no competing interests.

## Authors' contributions

IP carried out the part of work concerning InChIKey 2nd block and drafted the related text and introductory notes. AE carried out the computations, interpreted the results and drafted the text concerning InChIKey 1st block, while KB designed the related computational experiments and developed necessary software. AM, DT, and SH participated in the design and coordination of the study, as well as in drafting/revising the manuscipt. All authors read and approved the final manuscript.

## Supplementary Material

Additional file 1 **Contains supplementary material related to the first block testing.** Table S1 of this file presents an example of molecular formulae used with counts of generated structures and Figure S1 gives a structure generation software screenshot. The file also contains Table S2 which is a non-truncated version of Table 2 of the main body. Click here for file
