# Supplementary material for: InChIKey collision resistance: an experimental testing
Source: J Cheminform. 2012 Dec 20;4:39. doi: 10.1186/1758-2946-4-39 (PMC3558395; doi:10.1186/1758-2946-4-39)
Supplement: Additional file 1 — Contains supplementary material related to the first block testing. Table S1 of this file presents an example of molecular formulae used with counts of generated structures and Figure S1 gives a structure generation software screenshot. The file also contains Table S2 which is a non-truncated version of Table 2 of the main body. [file 1758-2946-4-39-S1.pdf]

Figure S1. Example of algorithmic definition of molecular formula range for structure generation in ACD/Structure Elucidator. The data define the range of molecular formulae $C_{2\text{-}6}H_{0\text{-}24}Cl_{1\text{-}3}F_{1\text{-}3}O_{0\text{-}3}$ and RDBE (Ring Double Bond Equivalents) range 0-2 that corresponds to 484 specific molecular formulae used in structure generation in this set.

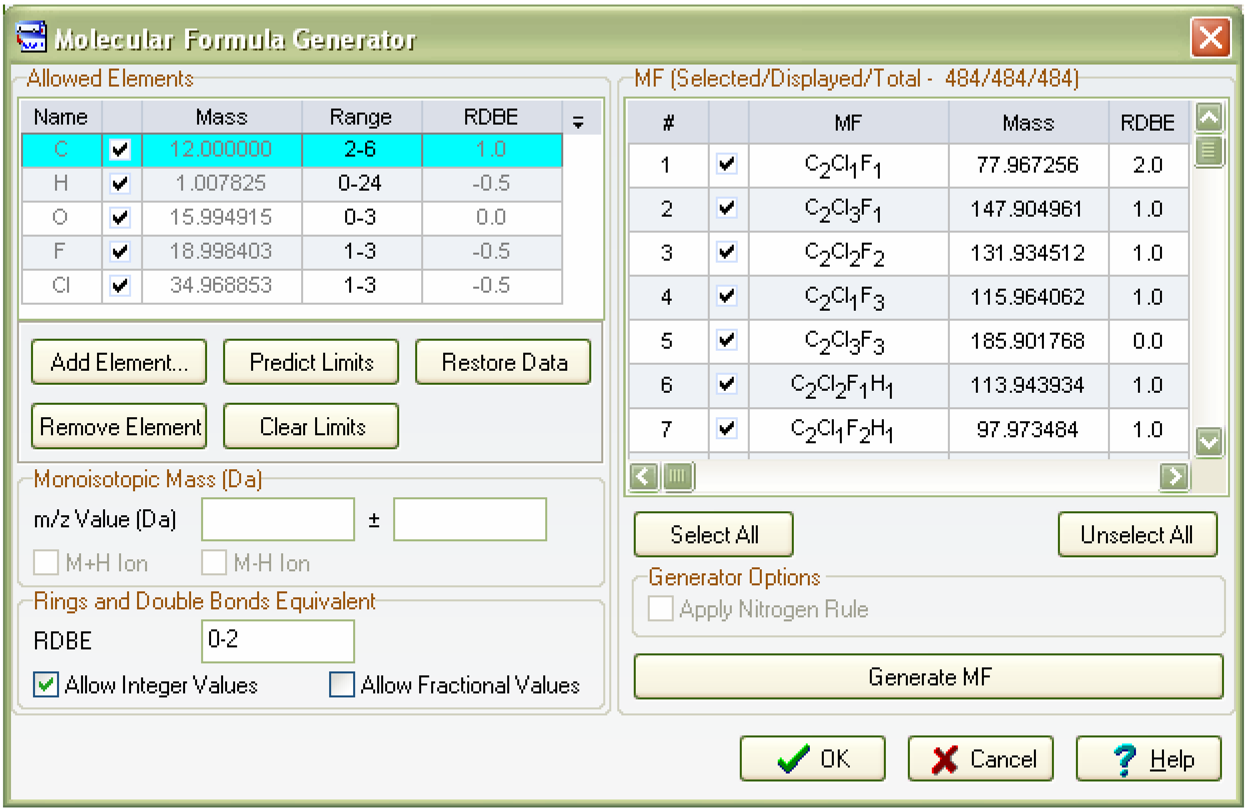

Table S1. The number of isomers generated for the compounds of molecular formulae in range $C_6H_{8\text{-}9}Cl_{1\text{-}3}F_{1\text{-}3}O_{0\text{-}3}$.

#	Formula	MW	RDBE	Number of isomers
1	C6H8F1Cl1	134.0299	2	1391
2	C6H8F1Cl3	203.9676	1	2702
3	C6H8F2Cl2	187.9971	1	3668
4	C6H8F3Cl1	172.0267	1	2702
5	C6H8F3Cl3	241.9644	0	3012
6	C6H8O1F1Cl1	150.0248	2	18104
7	C6H8O1F1Cl3	219.9625	1	36777
8	C6H8O1F2Cl2	203.992	1	50194
9	C6H8O1F3Cl1	188.0216	1	36777
10	C6H8O1F3Cl3	257.9593	0	42245
11	C6H8O2F1Cl1	166.0197	2	134456
12	C6H8O2F1Cl3	235.9574	1	283399
13	C6H8O2F2Cl2	219.9869	1	390235
14	C6H8O2F3Cl1	204.0165	1	283399
15	C6H8O2F3Cl3	273.9542	0	334098
16	C6H8O3F1Cl1	182.0146	2	731783
17	C6H8O3F1Cl3	251.9523	1	1595093
18	C6H8O3F2Cl2	235.9819	1	2211485
19	C6H8O3F3Cl1	220.0114	1	1595093
20	C6H8O3F3Cl3	289.9491	0	1928091
21	C6H9F1Cl2	170.0065	1	1348
22	C6H9F2Cl1	154.0361	1	1348
23	C6H9F2Cl3	223.9738	2	1494
24	C6H9F3Cl2	208.0033	2	1494
25	C6H9O1F1Cl2	186.0014	1	17726
26	C6H9O1F2Cl1	170.031	1	17726
27	C6H9O1F2Cl3	239.9687	2	20266
28	C6H9O1F3Cl2	223.9983	2	20266
29	C6H9O2F1Cl2	201.9964	1	133242
30	C6H9O2F2Cl1	186.0259	1	133242
31	C6H9O2F2Cl3	255.9636	2	156448
32	C6H9O2F3Cl2	239.9932	2	156448
33	C6H9O3F1Cl2	217.9913	1	734507
34	C6H9O3F2Cl1	202.0208	1	734507
35	C6H9O3F2Cl3	271.9585	2	884820
36	C6H9O3F3Cl2	255.9881	2	884820
**TOTAL**				**13 584 406**

Table S2. Number of InChIKeys containing various letters at the specific position of first block for a set of 998753 isomers with molecular formula $C_9H_8O_1$ (non-truncated version of Table 2 of the paper).

Position	1	2	3	4	5	6	7	8	9	10	11	12	13	14
**A**	41424	38195	37819	41079	38163	38223	41584	37995	38514	41357	38061	38319	50838	38899
**B**	41217	38051	38488	41389	38072	38485	41513	38084	38388	40757	37861	38578	50682	39406
**C**	41269	38190	38517	41184	37948	38439	41297	37723	38549	41181	37763	38376	50781	39005
**D**	41001	38206	38037	41580	37855	38360	41168	38238	38336	41234	38090	38485	51039	38854
**E**	0	37959	38651	0	38143	38340	0	37889	38438	0	38057	38177	50742	39086
**F**	41368	37881	38109	41273	38039	38453	41247	38292	38428	40631	38246	37991	51004	39064
**G**	41211	38055	38334	41190	38036	38452	41141	37933	38651	41049	38242	38209	50887	38874
**H**	41262	37943	37998	41162	37801	38369	41324	38210	38338	41324	38274	38502	50697	39151
**I**	41330	37739	38168	41354	38124	38463	41110	37906	38618	41140	37994	38479	50573	39028
**J**	40967	37946	38477	40784	38036	38427	41339	38171	38576	41236	37891	38246	50684	39019
**K**	41119	38238	38530	41291	38100	38092	41212	37770	38352	41461	37850	38900	50790	39227
**L**	41266	38067	37861	41248	38019	38485	41671	38065	38252	41211	37810	38352	50797	38981
**M**	41228	38068	38678	41046	38074	38626	41168	38144	38536	41453	38130	38213	50337	38896
**N**	41001	38069	38658	41005	37946	38241	40650	38361	38588	41435	37779	38069	50654	38998
**O**	41017	37920	38332	41154	38307	38548	41385	38330	38015	40966	37632	38710	50555	38902
**P**	41227	38280	38735	41008	38160	38360	41163	38371	38445	41105	38002	38330	50726	39038
**Q**	41247	37967	38513	41177	37575	38078	41455	38142	38154	41163	38658	38468	51092	39066
**R**	41280	37734	38436	41486	38141	38394	41077	38068	38508	41427	38204	38163	50340	39020
**S**	41174	38053	38501	41240	38045	38354	41245	37849	38291	41615	37805	38216	50686	36967
**T**	9923	38262	38535	9739	38222	38486	9514	38229	38588	9788	38312	38392	34849	37433
**U**	41390	39657	38696	41152	39532	38536	41034	39553	38206	41137	39806	38322	0	36874
**V**	41012	39788	38249	41019	39516	38546	41312	39609	38278	41331	39492	38460	0	36944
**W**	41184	39533	38739	41441	39767	38473	41159	39252	38324	41214	39552	38755	0	37225
**X**	41277	39600	38302	41026	39581	38485	41123	39670	38620	41213	39814	38887	0	36937
**Y**	40993	39475	38893	41431	39911	38562	40964	39375	38350	41318	39776	38563	0	37171
**Z**	41366	39877	38497	41295	39640	38476	40898	39524	38410	41007	39652	38591	0	36688

The data demonstrate that no InChIKey contains letter E at the positions 1, 4, 7, and 10; letters from U to Z at the position 13, and that letter T occurs four times less frequently at the positions 1, 4, 7, and 10.
